# Immune function and parasite resistance in male and polymorphic female *Coenagrion puella*

**DOI:** 10.1186/1471-2148-6-19

**Published:** 2006-03-07

**Authors:** Gerrit Joop, Andreas Mitschke, Jens Rolff, Michael T Siva-Jothy

**Affiliations:** 1 Zoologisches Institut, AG Ökologie, Technische Universität Braunschweig, Braunschweig, Germany; 2 Department of Animal and Plant Sciences, University of Sheffield, Sheffield, UK; 3 lnstitut für Mikrobiologie, Technische Universität Braunschweig, Braunschweig, Germany

## Abstract

**Background:**

Colour polymorphisms are widespread and one of the prime examples is the colour polymorphism in female coenagrionid damselflies: one female morph resembles the male colour (andromorph) while one, or more, female morphs are described as typically female (gynomorph). However, the selective pressures leading to the evolution and maintenance of this polymorphism are not clear. Here, based on the hypothesis that coloration and especially black patterning can be related to resistance against pathogens, we investigated the differences in immune function and parasite resistance between the different female morphs and males.

**Results:**

Our studies of immune function revealed no differences in immune function between the female morphs but between the sexes in adult damselflies. In an experimental infection females infected shortly after emergence showed a higher resistance against a fungal pathogen than males, however female morphs did not differ in resistance. In a field sample of adult damselflies we did not find differences in infection rates with watermites and gregarines.

**Conclusion:**

With respect to resistance and immune function 'andromorph' blue females of *Coenagrion puella *do not resemble the males. Therefore the colour polymorphism in coenagrionid damselflies is unlikely to be maintained by differences in immunity.

## Background

Colour polymorphisms are widespread and prominent in several taxa such as coral reef fish [1], birds [2] and tree frogs [3]. In many cases the genetic basis has been investigated [1,4]. However, the nature of selection pressures maintaining such polymorphisms are rarely known. One exception is a study on the pea aphid *Acyrthosiphon pisum*. In this species, both red and green colour morphs occur; these morphs differ in their susceptibility against natural enemies [5]. Polymorphisms can also be maintained by sexual selection, as suggested for plumage coloration in some birds [6] and in guppies [7]. Three major explanations have been proposed to explain the evolution and maintenance of such polymorphisms: frequency-dependent selection, heterozygote advantage [8] and environmental fluctuations. The last case has been elaborated by Reinhold [9]: fluctuating selection can maintain polymorphisms if the polymorphism is sex-limited.

Frequently, colour polymorphism is restricted to one sex as for example in the lizard *Uta stansburiana*. These lizard morphs follow different life history strategies and a trade-off between immunity and population density has been identified, such that the decline in antibody responsiveness with an increased number of neighbors is morph dependent [10,11].

In insects female colour polymorphisms are widespread among damselflies, such as polymorphism in female coenagrionids (Zygoptera, Odonata). Usually one female morph resembles the male, dubbed the andromorph, while one or more other "typical" female morphs are called gynomorphs [12]. This polymorphism has been shown to be heritable in at least three coenagrionid species [13-16] and is most likely to be the case in all coenagrionids.

Several hypotheses to explain the evolution and maintenance of female polymorphism in damselflies have been suggested. These explanations are, with few exceptions solely based on observations on mating behavior and most studies do not include any data on fitness or fitness components. Few studies have linked their results to the general theories for the maintenance of these polymorphisms [8,9,17] and even fewer have investigated correlations between coloration and other physiological or life history traits [18].

Broadly these damselfly-specific ideas fall into two categories: (i) the density dependent/male mimic hypothesis [12,19] or (ii) the learned mate recognition hypothesis (LMR) [[20] and references therein] or variations on them [e.g. [21]]. Coenagrionids have a non-territorial mating system with a high rate of male harassment. Copulations can last several hours and females are forced to mate several times, even though they could store enough sperm to fertilize their entire egg number from only one mating [22]. The male mimic hypothesis states that andromorphs mimic males to avoid male harassment with the risk of no matings under low population densities [19]. In contrast, according to the LMR idea males learn to recognize the most common morph in the population, which usually is the andromorph [[23], but see [24]] and therefore the less common morph can avoid harassment. Svensson et al. [25] recently showed that frequency-dependent selection maintains the polymorphism in the wild in *Ischnura elegans*. They combined extensive field sampling with a population genetic model and concluded that fecundity differences between the three female morphs are sufficient to maintain the polymorphism via frequency-dependent selection. Their analysis was based on the assumption that sexual conflict underpins the fecundity differences, however the sexual conflict was not demonstrated.

If female morph is correlated with other traits such as life history traits or physiological traits, than selection on these traits will affect morph frequencies. Fluctuating selection on such traits can be strong enough to maintain sex-limited polymorphisms [9]. This happens, because the disfavoured loci are shielded from selection in the mono-morphic sex. In damselflies morph differences in life history and related traits have hardly been studied. However, differences between female morphs in survivorship [26], and development time have been reported [18] and in other species such life history difference are reflected in differences in immune function. Here, we use the azure damselfly *Coenagrion puella *which has three different female morphs, an andromorph (blue), a gynomorph (green) and an intermediate (blue-green), as the study species. We propose that female colour polymorphism could be related to immune function and that selection on immune function and melanism could contribute to the maintenance of the colour polymorphism. There are five independent findings that make this hypothesis an interesting scenario to study, (a) *Coenagrion puella *shows a sex difference in investment in immune function during the larval stage [27] and in resistance to parasites [28]. *C. puella *females have a higher haemocyte load and a higher activity of the enzyme Phenoloxidase (PO), a common pattern in many insects [29].

Haemocyte load and PO are important components of insect immune function [30]. Both of these immune defense traits have frequently been linked to higher resistance in insects [31-33]. (b) Female *C. puella *have larger black cuticular patterning (melanin) on their abdomens when compared to males, however there are no differences between the female morphs [G. Joop, unpublished data]. The PO cascade is involved in the melanization and sclerotization of the cuticle [30]. This is particularly important for defense against fungal infections, as entomopathogenic fungi hydrolyze cuticular proteins [30]. (c) It has been reported that mating frequencies differ between the female morphs in several species [see references in [20]]. In the damselfly *Matrona basilaris japonica *it was shown that mating reduced encapsulation of artificial antigens in a wild population [34]. (d) Development time is related to investment in immune function in damselflies [35], such that faster development results in lower investment into immune function. Different life histories, such as morph specific survival rates [26] and development rates [18] are likely to select for different investment in immune function [29,36]. (e) Because immune function is the mediator of parasitic impact and wound repairs, it is likely to be an important indirect fitness trait [37].

Here we aim to elucidate the relationship between colour polymorphism and immune function in order to understand the maintenance of colour polymorphisms. We expect the female morphs to differ from males and differences between the female morphs are also likely. Specifically we investigated whether the three female morphs [38] and/or the males differ in (i) immunity in adults or (ii) resistance to a novel pathogen in adults infected after emergence (tenerals) and (iii) whether there is any indication for differential resistance in the field to two prevalent natural parasites.

## Results

### Immune parameters and condition

**Figure 1:**
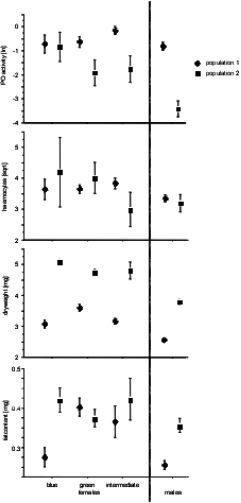
**Graphs illustrating the differences in immune parameters (two top panels), size (third panel from top) and condition (bottome panel) between males, blue (andromorph) females, blue green females and green females of *Coenagrion puella *in two populations**. Shown are means and standard errors.

We first analysed whether adult males and females differ in immune function and condition. A MANCOVA (Table 1) revealed that colour classes, males were treated as 'colour class' in this analysis, as well as populations differed in immunity and condition. The between subjects tests (Table 2) showed, that the populations differ only in condition but not in immune parameters. However, we found a significant interaction between population and colour class for PO activity. All female morphs group together and differ from the males (Table 3), except for dry weight and fat content, where the intermediate females differ neither to the other female morphs nor to the males. Overall, PO activity, haemocyte load, dry weight and fat content were always higher in females (Fig. 1). Subsequently we repeated the same analyses, but excluded the males. We did not find differences between female morphs in PO activity, haemocyte load, dry weight or fat content (p > 0.5 in all cases).

**Table 1:** MANCOVA (Pillai's trace) for immunity as measured by PO activity and haemocyte counts and condition for the different 'colour classes' (males, blue, bluegreen and green females) and populations in 2003, controlling for size (fatless weight).

**source**	**value**	**F**	**Hypothesis df**	**Error df**	**P**
'colour class'	0.147	3.870	9	675.00	< 0.001
population	0.127	10.783	3	223.00	< 0.001
'colour class' × population	0.095	2.457	9	675.00	0.009
fatless weight	0.972	2457.220	3	223.00	< 0.001

**Table 2:** Tests of between subjects effects for immunity as measured by PO activity and haemocyte counts and condition for the different 'colour classes' (males, blue, bluegreen and green females) and populations in 2003.

**source**	**dependent variable**	**df**	**F**	**P**
**'colour class'**	PO activity [ln]	3	7.535	< 0.001
	haemocytes [sqrt]	3	4.593	0.004
	dry weight	3	2.903	0.036
	fat content	3	2.903	0.036
**population**	PO activity [ln]	1	1.376	0.242
	haemocytes [sqrt]	1	3.226	0.074
	dry weight	1	28.106	< 0.001
	fat content	1	28.106	< 0.001
**'colour class' × population**	PO activity [ln]	3	3.048	0.030
	haemocytes [sqrt]	3	2.422	0.067
	dry weight	3	1.790	0.150
	fat content	3	1.790	0.150

**Table 3:** Pairwise comparisons of immunity as measured by PO activity and haemocyte counts and condition for the different 'colour classes' (males, blue, blue-green and green females).

**dependent variable**	**'colour class' (1)**	**'colour class' (2)**	**mean difference (1–2)**	**SE**	**P**
**PO activity [ln]**	blue female	green female	0.579	0.0531	0.277
		intermediate female	0.265	0.0568	0.641
		male	1.860	0.0542	0.001
	green female	intermediate female	-0.314	0.0395	0.428
		male	1.281	0.0364	0.001
	intermediate female	male	1.595	0.0405	< 0.001
**haemocyte [sqrt]**	blue female	green female	0.120	0.0431	0.781
		intermediate female	0.469	0.0462	0.311
		male	1.133	0.0441	0.011
	green female	intermediate female	0.348	0.0321	0.279
		male	1.013	0.0296	0.001
	intermediate female	male	0.665	0.0330	0.045
**dry weight [mg]**	blue female	green female	-0.028	0.045	0.533
		intermediate female	-0.046	0.048	0.338
		male	-0.106	0.046	0.022
	green female	intermediate female	-0.018	0.034	0.590
		male	-0.078	0.031	0.012
	intermediate female	male	-0.060	0.034	0.083
**fat content [mg]**	blue female	green female	-0.028	0.045	0.533
		intermediate female	-0.046	0.048	0.338
		male	-0.106	0.046	0.022
	green female	intermediate female	-0.018	0.034	0.590
		male	-0.078	0.031	0.012
	intermediate female	male	-0.060	0.034	0.083

**Figure 2:**
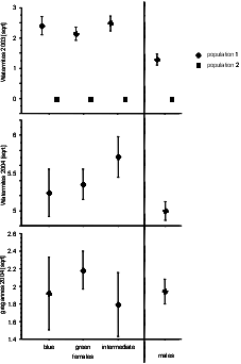
**Graphs illustrating the mean abundance of parasites on males, blue (andromorph) females, blue green females and green females of *Coenagrion puella *in two populations. The top graph shows water mite abundance in 2003, the panel in the middle shows the watermite abundance in 2004, and the bottom panel depicts the abundance of gregarines in 2004**. Means for natural parasite infection and standard errors are shown.

### Fungal infection experiment

**Figure 3:**
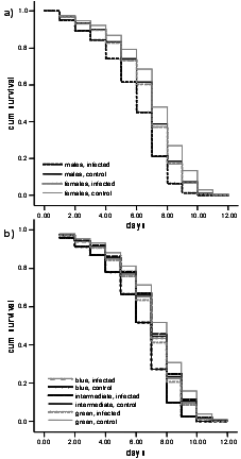
Survival of *Coenagrion puella *in two populations after fungal infection and for non-infected controls, a) comparing males and all females, b) comparing the different female morphs.

First we tested whether males and females differ in their survival, which is highly significant for sex (n = 378, Z = -3.84, p = 0.00012) and treatment (n = 378, Z = -4.67, p < 0.0001) (Fig. 3a), therefore fungal treatment reduced survival differently depending on sex, and females survived better. In a second analysis we tested for survival differences between the different female morphs (Fig. 3b). Again the fungal treatment has an influence on survival (n = 159, Z = -2.263, p = 0.024) but between the female morphs no significant differences were found (n = 159, Z = -0,998, p = 0.320). In a third model we used treatment and colour morph including males as covariates. Both covariates were significant (n = 378, colour morph: Z = -3.72, p = 0.0002, treatment: Z = -4.53, p < 0.0001). Subsequently we compared the first model (only gender differences) with the third model (that included female colour morphs and males). However, we did not find a difference in the performance of these models (Difference in AIC between model 1 and 3: as this is >2, the models do not differ). The differences in general colour morph in model 3 are explained by the differences between males and females alone.

### Natural parasites

We did not find a difference in water mite or gregarine abundance for the 'colour classes' (note that males were entered as colour class in the statistical model) (Table 4). Water mite infestation differs between the populations in 2003 with hardly any water mites present in population 2. In general, infestation with these parasites seems not to be very high and with only slight differences for water mites between 2003 and 2004 (Fig. 2).

**Table 4:** Analyses (ANCOVAs) of the mean numbers of water mites in 2003 and 2004 and for the mean number of gregarines in 2004 in *Coenagrion puella *males and females (coded as colour class). In all three analyses body size was accounted for by using head width as a covariate.

**Year and parasite**	**source**	**df**	**F**	**P**
**2003, water mites**	**'colour class'**	3	0.605	0.612
	**population**	1	12.201	0.001
	**'colour class' × population**	3	1.886	0.682
	**head width**	1	8.397	0.004
**2004, water mites**	**'colour class'**	3	2.261	0.082
	**head width**	1	11.428	0.001
**2004, gregarines**	**'colour class'**	3	0.419	0.740
	**head width**	1	0.024	0.877

## Discussion

We found significant differences between adult male and female *Coenagrion puella *from the wild, with females having a stronger immune function as indicated by the higher haemocyte counts and PO activity. Moreover, teneral females were more resistant against fungal infection with *M. anisopliae *than males. However, we did not find any such differences between the female morphs. We found no evidence for any differences in parasite abundance in the field, neither between the sexes nor within the female colour morphs. Why do females invest more in resistance and immunity? And why are there no differences between the female colour morphs?

Adult females have generally a higher PO activity and a higher haemocyte load than the adult males in *C. puella*. Therefore our results support the data of Joop and Rolff [27], where the sexes differed in their investment in immunity immediately after emergence in *C puella*. This also shows that the sexual dimorphism is maintained later in the adult life. The differences in immune function were also reflected in the higher resistance of teneral females against the fungal pathogen. Female *C. puella *have a higher black melanin content in their cuticle than males (G. Joop unpublished data). Black patterning of insect cuticle is commonly a product of melanin, a pigment, that has antimicrobial properties and which is produced via the PO cascade [30,32]. Wilson et al. [39] have shown that melanic moths exhibit a higher resistance towards entomopathogenic fungi than non-melanic individuals. *M. anisopliae *and most other entomopathogenic fungi of the order Cordyceps have spores that produce a lysozyme which dissolves the insect's cuticle [40]. The spores can then enter the haemocoel and parasitise the host [41]. The fact that all female morphs invest more in PO than males, which is most likely involved in resistance against the fungal infection [30], make a trade-off between PO activity and cuticular melanization unlikely. It was recently shown that PO responds in positive correlated fashion to selection on cuticular darkness in the mealworm beetle [42]. It could be speculated that in other coenagrionid damselfly species such as *Ischnura elegans*, where males and females are almost similar in black patterning, no such differences in fungal resistance occur. If this holds true, it would constitute a potential cost of being a nearly perfect male mimic in *C. puella*: females would forfeit their higher resistance by decreasing their black melanin content.

The differences in immune function and resistance between the sexes can be explained by differences in life-history, males increase their fitness by increasing their mating rate while females increase their fitness by longevity [29,43-45]. Furthermore females have a much higher investment in reproduction than males, as egg production is much more energy consuming than the production of sperm [46]. Because females achieve higher fitness through longevity and pay higher costs for reproduction, they should invest optimally in immunity as well to ensure a long reproductive life.

An additional selection pressure on black patterning may be found in thermoregulation. The mortality of mitosporic fungi such as *M. anisopliae *has been shown to be highly dependent on the environmental temperature and therefore the insect's body temperature [47,48]. Locusts use this and actively seek places with higher temperature after fungal infection in order to overcome it [48]. Male *C. puella *spend most of their time near the water waiting for females, while females spend more time searching for prey and resting in hedges [49]. Therefore males are probably exposed to higher temperatures than females. The higher black content might help the female to gain higher body temperature while being exposed to the sun. Furthermore, it has been suggested that male *C. puella *are capable of thermoregulatory colour change [38]. This thermoregulation is assumed to protect flight muscles and sperm from overheating. Therefore it could be, that female cuticle does not simply have a higher black melanin content, but that reduced melanin patterning in males is adaptive to avoid overheating. With the more complex structure of the female cuticle due to the higher melanin content, the cuticle might also give additional UV protection but certainly more data on this would be needed.

Blue females are dubbed andromorphs [12] and also are supposed to mimic male behaviour to avoid male harassment [12,50-52], why did we find no differences in immune defence between the female morphs? As all females have the same reproductive requirements in terms of for example egg production and oviposition, this might outweigh any behavioural differences between the morphs. Studies in related coenagrionid species have reported differences in life histories such as survival [26] and development time [18], traits that have been shown to be linked to immune function in other studies [35] and here with respect to gender differences in resistance against fungal infections. Contrary to our expectations the supposed different mating tactics in the female morphs do not seem to mirror different immune strategies, as reported in the female polymorphic lizard *U. stansburiana *[11]. Longevity should of course be the overriding factor, but it seems that this is not achieved by different strategies involving immunity and resistance. It is noteworthy, that we sampled adult individuals in the wild. Therefore differences in survival selection during the pre-reproductive stage could potentially obscure differences in immune function, if survival and immune function during this stage are correlated. Further studies are needed to disentangle these possibilities and to account for potential morph differences in plasticity of immune function as well. An indicator for plasticity in immune function in the wild is our result that shows an interaction between colour class and population for PO activity. We have no data that explain these differences, possible explanations include genetic differences between the populations or different diets.

From a broader perspective this is supported by our results for parasitism in the wild. We found no significant differences overall for water mite or gregarine load. Taking into account the fact that all damselfly larvae (both sexes and all female morphs) hatch and move to more shallow areas of the pond inhabited by potentially infecting water mites, it is not surprising that we found no differences in parasite load between gender or morphs [53]. Gregarines are ingested with the food, either in the larval or adult stage. And as long as the food infected is equally distributed over the population's habitat and they eat the same food types, it is not surprising that no differences in parasite load were found. As a next step it should be studied whether the parasites differ in their effect on the female morph's longevity: Braune and Rolff [28] demonstrated that the males and females *C. puella *differed in their survival, which was also dependent on water mite load. Furthermore females need to obtain more food because of the expenditure upon egg production. Therefore females have an increased risk of gregarine infection. In other damselfly species it has been shown that PO activity is positively correlated with resistance against gregarines [54] and that gut and haemolymph PO activity is positively correlated [55]. Even though we have no data on this link in *C. puella*, one possible explanation is that females have a higher gregarine infection risk, yet because of their higher investment in immunity this is not reflected in the abundance of gregarines in hosts in the wild.

## Conclusion

In conclusion, our results do not support the idea that blue females of *C. puella *are male like with respect to immunity and resistance. This is probably due to the selection pressure upon female morphs to have the same reproductive level. Differences in immune function are unlikely to underpin fluctuating selection on polymorphism in the wild *sensu *[9], unless morph differences in life history [18] translate in to differences in immune function in the wild given the tenet that immunity in damselflies is plastic [27,35]. It is likely that the female morphs have different costs associated with maintaining this standard level of reproduction and therefore have to trade-off elsewhere, e.g. in clutch- or egg size, especially as reproduction seems to be of overriding importance.

## Methods

### Study organism

*Coenagrion puella *(Zygoptera, Odonata) is a common and well studied species [27,28,44,45] of northern and central Europe, usually occurring in small ponds [56]. Larvae hibernate in later instars. Shortly before emergence the damselfly larvae move to shallow water regions, where water mites might settle onto them, while they only later attach to the adult and start parasitizing [57]. As damselflies keep on feeding after emergence they may become infested with endoparasites as well e.g. eugregarines, as has been shown for several other damselfly species [e.g. [54,58]]. These endoparasites attach to the mid-gut epithelium, thus rupturing or blocking it [60]. After adult emergence it is easy to distinguish the sexes but not the female colour morphs, as the individuals are not yet coloured. Becoming fully coloured and sexually mature takes, depending on weather conditions, seven to ten days, however the colour morph is detectable after two to three days. For *C. puella *three female colour morphs are described [38] an 'andromorph' (blue), a 'gynomorph' (green) and an 'intermediate' morph (blue-green).

### Immune parameters and condition

Damselflies were collected as adults from two different populations near Braunschweig, Lower Saxony, Germany, over the whole flight season 2003. Population 1 is a well established population of high density and older than 15 years while population 2 is not older than three years and is of lower density [Joop, pers. obs.]. The populations are 9 km apart, separated by a motorway and therefore almost certainly far enough to prevent gene flow. We used only fully coloured and therefore sexually mature animals but made sure that they were not too old by checking their wings for damage and stiffness [11,22]: those with damaged wings were excluded. Furthermore animals were checked for scars resulting from previously attached water mites If there were scars but no water mites the damselfly has mated at least once (*Arrenurus *water mites detach during oviposition, [57]) and they were excluded from the analysis.

### Immune parameter

As immune parameters we measured PO activity (humoral immunity) and counted haemocytes (cellular immunity), for both we followed the protocol of Joop and Rolff [27]. To obtain the haemolymph, the damselfly's thorax was perfused with 0.3 ml sodium cacodylate buffer (0.01 M Na-Coc, 0.005 M CaCl_2_). 20 μl of the resulting solution was pipetted into one well of a multiwell slide (adhesive epoxy-coated 12-well slides, Roth L209.1) to estimate the number of haemocytes. Haemocytes were stained with DAPI and counted using and image analysis system as described in [35].

The rest of the haemolymph sample was frozen at -80°C to disrupt cell walls and stop enzyme function. To measure PO activity 40 μl of the frozen haemolymph sample was defrosted and mixed with 100 μl distilled water, 20 μl phosphate buffered saline (PBS) and 40 μl I-DOPA (4 g/ml) as the substrate in 96 well cell culture plates. Samples were measured every 15 seconds over 30 minutes at 30°C and at a wavelength of 490 nm. The slope of the reaction was calculated using softmax pro software and V_max_, the velocity of maximum substrate conversion, was recorded.

### Condition

We measured dry weight and fat content as correlates of condition. Both have been shown to be good estimates of condition in *C. puella *[60]. As females store the eggs in their abdomen and therefore have a higher weight and fat content in this compartment, we only analysed the condition parameter for the thorax in order to have a better comparison between the sexes. Measurements were taken according to Joop and Rolff [27] and to correct for size, fatless weight was used.

### Statistical analyses

As our estimates for immune function and condition were highly correlated these data were analysed using a MANCOVA with fatless weight as covariate. Fatless weight is a better correction for size than head width and therefore should be used whenever possible. Number of haemocytes (square root transformed [61]), PO activity (In-transformed [61]), dry weight and fat content were used as dependent variables, 'colour morph' (male; blue, green or blue-green females) and population (1 or 2) as factors. The MANCOVA was followed by tests of between-subjects effects and pairwise comparisons. All analyses were performed using SPSS 12.0.1.

### Fungal infection experiment

Damselfly larvae were caught in the first two weeks in April 2004 in the area "Klei" near Braunschweig (Lower saxony, Germany, 52°21'N, 10°35'E), when they were in the final three instars. Larvae were randomly placed into one of eight tanks (length:width 36:30 cms, filled with 18 litres of de-chlorinated water). Fifty larvae were placed in each tank. Plastic mesh was provided as a climbing structure, allowing the larvae to leave the water for hatching. Tanks were a priori assigned to control or fungal treatment. Tanks were placed in a temperature controlled room at 16°C and a light-cycle of 12:12 h. All damselflies were held under the same feeding conditions. Tanks were monitored every morning for eclosing individuals, the first adult damselflies were found on 17^th ^June 2005.

As a fungal pathogen we used *Metarrhizium anisopliae*, which is known to be an entomopathogenic fungus [62]. Spores of *M. anisopliae *attach to the insect's cuticle and grow through the cuticle. Several fungi are known to attack damselflies [40,63], but *M. anisopliae *is not a natural pathogen of *C. puella*. We chose this novel pathogen because we were interested in resistance and not host-parasite co-evolution.

### Spore suspension of *M. anisopliae*

*M. anisopliae *(strain F142) was grown on PDA (Potato Dextrose Agar, Merck) and later on 2 % biomalt agar for better sporulation at 25°C in petri dishes. To obtain the hydrophobe spores a 0.05 % Triton × 100 suspension was pipetted onto the petri dish, spores wiped off and their concentration adjusted using a Thoma haemocytometer [64]. Spore concentrations were between 1.4 × 10^7 ^and 3.0 × 10^7 ^spores per ml. Spore suspensions were stored in the fridge for no longer than 24 h before use [41].

### Inoculation with *M. anisopliae*

Imaginally eclosed damselflies were collected every morning. Their head width as a measure of size [45] and the fresh weight were recorded. The abdomen of control individuals was dipped in microcentrifuge tubes each containing 1.5 ml Triton-X-100, while treatment individuals were dipped in 1.5 ml spore suspension (in Triton-X-100). Adult damselflies were held in 500 ml plastic containers with wet filter paper to provide humidity. Containers were closed with gauze. To prevent cross-infection inoculated treatment damselflies were kept in a separate 1 m^3 ^glass cube. To have optimal starting conditions for the spores to grow, all animals were kept for 8 h at 27°C [64]. Thereafter damselflies were kept at 16°C, which is the ideal temperature to keep them alive as long as possible without feeding [Joop, unpublished data]. Under bad weather conditions damselflies can survive in the wild without hunting for several days [20] so our experimental conditions are not unnatural in this respect. Furthermore under bad weather conditions, especially rainy but not too cold (above 10°C) weather fungal growth is higher [62] and therefore damselflies are most likely to struggle with fungal infections. Survivorship was recorded daily. Dead damselflies were collected and their surface sterilized using 70% Ethanol and sterile distilled water. These damselflies were subsequently put into a sterile petri dish with humid filter paper and checked after 2, 4 and 6 weeks for fungal growth and whether this was *M. anisopliae*. This was necessary to make sure that the fungal infection worked and to control the control individuals for fungal growth as well.

### Statistical analyses

Survival was analysed using a Cox regression as implemented in R [65]. As all animals died our data are non-censored. Days to death were used as a time estimate and treatment (control, infected) and 'colour morph' (male; blue, green or blue-green female) or sex (male, female) as co-variates. The performance of the models is compared using AIC [65].

### Natural parasites

Water mites are known to be common ectoparasites of damselflies, of which *Arrenurus cuspidator *is the most common in *C. puella *at least in one of our study populations [53]. They only parasitze adult individuals but attach in the larval stage [57]. In order to get an accurate estimate of the abundance of parasitic water mites we counted the parasitizing water mites on the damselflies collected as above for the two different populations in 2003. In 2004 water mites were only counted for population 1. Damselflies that showed scars were excluded, as the number of parasitic mites could not be estimated accurately. However, this is also another indicator of age, as they have to have mated before (see [57] and references therein).

Other common parasites in damselflies are gregarines [54], which parasitise in the mid-gut. Gregarines have so far not been described for *C. puella*. Therefore we collected age-controlled (see above) individuals of *C. puella *from population 1 in 2004 and dissected their mid-gut [see [54]]. In the dissected animals water mite numbers were also counted.

### Statistical analyses

To control for a potential relationship between body size and parasite abundance, head width was measured and included into analyses, of both, water mites and gregarines, as a covariate. We found no correlation between the numbers of different parasites and therefore analysed them separately using ANCOVAs with number of parasites (square root transformation yielded a distribution sufficient to use ANOVA [61]) as the dependent variable and colour morph and population (2003 data only) as factors. All analyses were performed using SPSS 12.0.1.

## Authors' contributions

Animals were collected and reared in the lab by GJ. The immune assays were performed by GJ, while AM adapted the fungal infection experiment to damselflies and conducted the classical and molecular microbiological methods to control for cross-infection in this experiment. JR and MSJ participated in design and coordination of the study. GJ and JR analysed the data and wrote the paper, JR revised the paper.
